# Bioremediation of Olein Industry Waste by *Cytobacillus* sp. OWB‐43 Strain

**DOI:** 10.1111/1758-2229.70424

**Published:** 2026-10-08

**Authors:** Julián Escalante, Nicolás Lencina, Ainelén Piazza, Paula Burdisso, Mario O. Salazar, Ricardo L. E. Furlan, Natalia Gottig, Jorgelina Ottado, Betiana Garavaglia

**Affiliations:** ^1^ Instituto de Biología Molecular y Celular de Rosario, Consejo Nacional de Investigaciones Científicas y Técnicas (IBR‐CONICET) and Facultad de Ciencias Bioquímicas y Farmacéuticas Universidad Nacional de Rosario (UNR) Rosario Argentina; ^2^ John Innes Centre Norwich Research Park Norwich UK; ^3^ Plataforma Argentina de Biología Estructural y Metabolómica (PLABEM) Ocampo y Esmeralda Rosario Argentina; ^4^ Área Farmacognosia, Facultad de Ciencias Bioquímicas y Farmacéuticas – Universidad Nacional de Rosario (UNR) Concejo Nacional de Investigaciones Científicas y Técnicas (CONICET) Rosario Argentina

**Keywords:** aerobic treatment, bioremediation, olein waste, phytotoxicity

## Abstract

The disposal of industrial vegetable olein waste poses a significant environmental challenge. In the present study, we investigated the bioremediation potential of *Cytobacillus* sp. OWB‐43 to treat a waste generated from vegetable olein production, a by‐product of sunflower, soybean, corn, and peanut oil industries with a chemical oxygen demand (COD) of 160,000 mg L^−1^. *Cytobacillus* sp. OWB‐43 decreased the COD in 18.75% (~30,000 mg L^−1^) and soluble solids in ether in 24.8% (7.2 g L^−1^). Moreover, maximum growth in population under stirring and static conditions was 3.7 × 10^8^ CFU mL^−1^ and 1.5 × 10^9^ CFU mL^−1^, respectively. Besides, bacterial treatment (both agitation and static), reduced phytotoxicity of the residues, as observed in both lettuce seedlings and onion bulbs, used as biosensors of toxicity. Furthermore, we demonstrated that treated residues can be safely disposed on soil without harmful effects. This statement was observed in soil irrigated with *Cytobacillus* sp. OWB‐43‐treated residue, which supported lettuce growth, with fresh and dry weight values comparable to those grown in water‐irrigated soil. While genera such as *Bacillus* and *Pseudomonas* are known for oily wastewater treatment, this study identifies *Cytobacillus* sp. as a promising and previously unreported bioremediation agent on olein waste treatment.

## Graphical abstract

*Cytobacillus* sp. OWB‐43 can bioremediate residue provided from edible oil refining. The bacterium reduces toxicity, chemical oxygen demand, and soluble solids, enabling safe disposal and supporting plant growth—offering a sustainable solution for waste from sunflower, soybean, corn, and peanut oil industries.

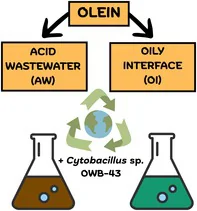

## 1 Introduction

Worldwide, vegetable oil production is an important industrial activity in several countries, with Indonesia, China, Malaysia, the USA, Brazil, and Argentina being the most prominent (https://www.nationmaster.com/nmx/ranking/vegetable‐oils‐production). In Argentina, the Gran Rosario Metropolitan is notable for its high concentration of oil production.

Edible oil refining generates large amounts of wastewater and solid residues that represent major environmental and disposal challenges. Crude vegetable oils contain triacylglycerides (TAGs), phospholipids (PLs), free fatty acids (FFAs), metals, proteins, and other minor compounds that must be removed to improve oil quality and stability (O'Brien 2008; Sampaio et al. 2015; Hails et al. 2020). Refining is necessary to remove undesirable impurities that affect the quality and shelf life of refined oils (Sonawane and Waghmode 2023). Most industrial plants still rely on chemical/alkali refining processes, which produce substantial waste streams enriched in soaps, oils, phospholipids, organic matter, and acidic effluents (Rajkumar et al. 2010; Sonawane and Waghmode 2023). During alkali neutralisation, FFAs are converted into soap stock, a complex mixture containing water, residual oil, phospholipids, proteins, and mucilaginous compounds (Fazli et al. 2013). Subsequent acidulation generates acid oil as a recoverable by‐product, olein, but also produces acid wastewater (AW) and oily interface (OI), residues characterised by acidity, oil content, and suspended colloidal material (Rajkumar et al. 2010) (Figure S1). These wastes cannot usually be discharged directly into water bodies due to regulatory restrictions, requiring additional physicochemical or biological treatments (Pandey et al. 2003). Alternative approaches such as physical or enzymatic refining can reduce oil losses and partially decrease waste generation, particularly during degumming (Elena et al. 2017; Cerminati et al. 2017, 2019; Sein et al. 2019; Hails et al. 2020). However, these methods still generate effluents with compositions similar to those produced by conventional refining, highlighting the persistent lack of efficient and sustainable treatment strategies for these industrial residues.

Bioremediation harnesses the metabolic potential of microorganisms to eliminate hazardous pollutants (Zabermawi et al. 2022) and may be applied to treat oily wastewater. This process involves the conversion of pollutants into harmless by‐products through microbial activity. Factors like temperature, pH, oxygen levels, and others influence this activity. Bioremediation is preferred due to its cost‐effectiveness, eco‐friendliness, and sustainability. Its effectiveness in the removal of pollutants from oily wastewater has been recently reviewed (Adetunji and Olaniran 2021). Both composting and landfarming are efficient and cost‐effective solid‐phase processes for degradation of organic compounds with minimal environmental impact. However, it presents some operational limitations, including high space requirements, time‐intensive processes, depth restrictions, and dependence on weather conditions, among others (Macci et al. 2021; Lukić et al. 2017). Strategies to accelerate biodegradation in solid phase include biostimulation and bioaugmentation (Bala et al. 2022). The first one involves the stimulation of the indigenous microorganisms (Omokhagbor Adams et al. 2015), while the second one involves the inoculation of specific bacterial strains into the soil, as an in situ bioremediation strategy (Mrozik and Piotrowska‐Seget 2010; Guarino et al. 2017). Although these techniques have proven successful, treatment times remain too long to meet current demand (Picado et al. 2001; Besalatpour et al. 2011; Guarino et al. 2017; Bala et al. 2022). In this context, studies on novel microorganisms and adoption of new technologies as alternatives to existing ones constitute promising strategies for oily wastewater treatment.

In particular, AW is periodically extracted from the pools and managed through landfarming, involving regular irrigation of soil to ensure proper treatment (Azubuike et al. 2016). However, the prolonged treatment time, mainly due to the residue's acidity, which affects the development of indigenous microorganisms, results in prolonged immobilisation of large land areas. Regarding OI, it remains accumulated in pools without treatment. The high hydrophobicity present in the sample creates significant bottlenecks for landfarming and solid‐phase bioremediation, primarily due to mass transfer limitations, low bioavailability, and strong adsorption to the soil matrix. Because microorganisms thrive in the aqueous phase, contaminants that do not dissolve in water remain completely out of reach for biological degradation (Rusănescu et al. 2025).

An *ex‐situ* bioremediation strategy that includes efficient microbial growth under controlled conditions is the utilisation of bioreactors (Jeon and Madsen 2013; Quintero et al. 2007; Tekere 2019; Bala et al. 2022). This procedure offers flexible design that can be adapted to different applications and purposes (Gargouri et al. 2011; Chikere et al. 2012; Azubuike et al. 2016; Pino‐Herrera et al. 2017). In bioreactors, simple or mixed microbial cultures are used, whereas contaminated environments can provide sources of microorganisms that are resistant or acclimated to pollutants (Lladó et al. 2013).

To address the environmental challenge of bioremediating the waste generated by the olein industry, we implemented an *ex‐situ* bioremediation strategy aimed at degrading and transforming this waste to allow its safe disposal in soil. To expand the current knowledge of microorganisms capable of treating with high efficiency a specific substrate poorly underexplored as oily wastewater, the goals of this work were to: (i) isolate and (ii) identify bacterial strains with surfactant‐producing capability and specific enzymatic activities that enable degradation of compounds in an olein waste mix (OWM) composed of AW and OI; (iii) analyse their ability to grow in OWM; (iv) reduce chemical oxygen demand (COD), soluble solids in ether (SSE), and phytotoxicity; (v) select the most promising strain/s for bioremediation according to their performance; (vi) employ it/them in a batch bioremediation strategy under both agitation and static conditions; and (vii) assess changes in the composition of OWM before and after treatment. For this purpose, bacterial isolates showing the highest residue treatment efficiency and the lowest phytotoxicity were selected to manage the residue prior to its final disposal in soil. Our approach not only facilitates the safe disposal of olein waste in soil but also provides insights into the broader application of bioremediation techniques for industrial waste management and their potential for sustainable waste treatment.

## 2 Experimental Procedures

### 2.1 Sample Collection, Physicochemical Characterisation and Bacterial Isolation

Residual samples from the olein industry were collected with wide‐mouth glass bottles washed with soap, rinsed with water, and finally rinsed with acetone (Baird et al. 2017). Samples, AW and OI, were provided by a company located in San Lorenzo department, in the province of Santa Fe, Argentina (32.830968 S 60.735907 W). According to the regulations of the Provincial Decree 2151/2014 (https://www.santafe.gov.ar/index.php/web/content/view/full/185309), wastewater used here is considered non‐hazardous industrial waste. Collected material used to analyse, cultivate, and isolate bacteria was preserved at 4°C. Conductivity and pH were measured using a Horiba U‐50 multiparameter device. Moreover, nearly all the elements of the periodic table were analysed by inductively coupled plasma mass spectrometry (ICP‐MS). Auxiliary equipment for the digestion of samples (microwave oven) was provided by the service (Center of Photosynthetic and Biochemical Studies (CEFOBI‐CONICET‐UNR)). To evaluate the concentration of chloride, nitrite, and sulfate anions, commercial detection kits from GT Lab were used. Nitrates were quantified using a HORIBA‐LAQUA‐Twin nitrate selective electrode with a detection range of 30–9900 ppm. In all cases, quantifications were performed in triplicate, and the average values and standard errors were calculated. Total solids analysis was conducted using an adaptation of a previously described protocol (Hamilton and Zhang 2016). Briefly, 1 mL of OWM was transferred into oven‐dried glass beakers pre‐weighed using an analytical balance (Mettler AE 163), and the initial mass was immediately recorded. The beakers containing the samples were then oven‐dried at 103°C for 24 h, after which the final mass was recorded. Total solids were calculated from the difference between the mass of fresh and dehydrated samples. All measurements were performed in triplicate, and the mean value and standard error were calculated.

For the isolation protocol, first, direct plating from AW and OI was carried out and thereafter, an enrichment strategy was performed (Figure 2). Briefly, the pellet obtained after centrifugation of 10 mL of AW for 5 min and 0.1 g of OI were resuspended in 100 μL of 0.9% (w/v) NaCl, plated onto Luria Bertani (LB) agar plates (Sambrook et al. 1989) and incubated at 30°C. Colonies that grew were then streaked onto fresh LB plates until single colonies were obtained. For the enrichment strategy, 1 volume of OI was mixed with 2 volumes of AW and adjusted at pH 7 (OI: 2AW). The suspension was incubated at 30°C with agitation at 180 rpm for 2 days. After incubation, serial dilutions were prepared in 0.9% (w/v) NaCl, and 100 μL aliquots were plated on LB agar as described above. Pure colonies were grown in LB medium until stationary phase and aliquots of these cultures were supplemented with 20% glycerol and frozen (−70°C) for storage. Moreover, bacterial growth was evaluated after incubation for 7 days at 30°C on 2% (w/v) agar plates formulated by olein waste mix diluted by half in tap water (OWM:H_2_O) at pH 7.0, regulated by using 4% (w/v) building lime, or without pH regulation. Bacterial growth was evaluated on 96‐well microplates in LB at 30°C for 15.5 h and was followed by measuring their optical density at 600 nm (OD600nm) using a Synergy 2 Reader, BioTek. Growth curves were performed using four values for each strain at each time point. The equation for fitting the growth curve at the exponential growth phase was: lnN = lnN_0_ + μt, in which N is OD_600nm_ at a given time, N_0_ is the initial OD_600nm_ at which growth becomes exponential and μ is the growth rate.

### 2.2 Enzymatic Activity Measurements

Protease activity was evaluated in LB medium supplemented with 2% (w/v) skimmed milk powder and 1.5% (w/v) agar was used. Inoculated plates were incubated for 48 h at 30°C. Protease‐secreting bacteria were evidenced by the appearance of halos of milk protein lysis around the colonies (Larsen et al. 1998). To evaluate lipase activity, tween agar medium (10 g L^−1^ peptone, 5 g L^−1^ NaCl, 0.1 g L^−1^ CaCl_2_·2H_2_O, 1% (v/v) Tween 20) was used. Inoculated plates were incubated at 30°C for 5 days. The appearance of a white precipitate, due to the action of lipase releasing fatty acids that precipitate as calcium salts around the colonies, was considered a positive result (Al Mohaini et al. 2022). Lecithinase production was determined by streaking LB‐agar plates supplemented with 5% (v/v) egg yolk. These plates were incubated for 48 h at 28°C. In this culture medium, a halo or precipitate appears around the lecithinase‐producing bacteria as a result of the hydrolysis of egg yolk lecithin (Lonon and Hooke 1991). For the qualitative analysis of cellulase activity, a protocol was adapted using LB‐agar supplemented with 1.5% (w/v) carboxymethylcellulose (CMC). Inoculated plates were incubated at 30°C for 5 days. At the end of the incubation period, an aqueous solution of Congo red (1% (w/v)) was added and left to act for 15 min. The solution was then removed, and the plates were washed with 1 M NaCl for 15 min. Cellulase activity was evidenced by a clear zone (Grata 2020). For surfactant production evaluation, bacterial strains were cultured in liquid LB medium at 30°C at 180 rpm until the exponential growth phase. Aliquots of 100 μL were then centrifuged at 5000 rpm for 6 min, the supernatant discarded, and the cell pellets resuspended in 10 mL of LB medium. These subcultures were incubated at 30°C and 180 rpm until reaching late exponential phase, after which they were centrifuged at 5000 rpm, and the resulting supernatants were collected for analysis. Surfactant production was evaluated using the droplet collapse technique (Bodour and Miller‐Maier 1998). Briefly, 96‐well plastic plates were incubated at 28°C for 16 h with shaking at 130 rpm, with 2 μL of mineral oil added to each well to ensure uniform coating. Subsequently, 2 μL of each culture supernatant were deposited onto the oil layer, forming droplets. The presence of surfactants reduces surface tension, resulting in an increase in droplet diameter proportional to surfactant concentration. Supernatant from *Pseudomonas aeruginosa* PA01 grown under the same conditions was used as a positive control, while LB medium without bacterial growth was used as a negative control. Droplets were examined using an Olympus MVX10 microscope, imaged with an Olympus DP72 camera, and droplet diameters were measured using Olympus DP2‐BSW software. The diameters obtained were compared with those of the controls to determine surfactant production in the evaluated samples.

### 2.3 Molecular Identification and Genome Sequencing of Cultured Bacteria

Molecular identification was carried out as described previously (Piazza et al. 2019). Briefly, single colonies were picked and suspended in 50 μL of sterile distilled water and heated at 95°C for 10 min. Aliquots of 5 μL were taken as a template to carry out 16S rRNA gene PCR amplification using the primers pair FD1 and RP1 (Clarridge 2004). The amplification products (1550 bp) were purified with Wizard R SV Gel and PCR Clean‐Up System (Promega) and were sequenced by capillary electrophoresis sequencing (CES) at Macrogen (Korea). For 16S rRNA gene, both strands were sequenced, and reverse sequences were transformed into reverse complement sequences using sites available on the web. These sequences were aligned with forward sequences using Clustal Omega (Sievers et al. 2011). EzBioCloud, a database that holds the taxonomic hierarchy of the Bacteria and Archaea, was used to classify bacterial isolates (http://www.ezbiocloud.net) (Yoon et al. 2017).

Furthermore, bacterial identification was carried out directly using Matrix Assisted Laser Desorption Ionisation‐Time Of Flight Mass Spectrometry (MALDI‐TOF MS) as previously described (Piazza et al. 2019). This technique allows bacterial identification at the species level by matching detected bacterial ribosomal protein masses to a reference database (Dingle and Butler‐Wu 2013; Welker and Moore 2011).

For bacterial genome sequencing, RNA‐free genomic DNA samples from OWB‐10, OWB‐30, OWB‐41 and OWB‐43 strains were isolated with EasyPure Bacteria Genomic DNA Kit (TransGen) and library preparation and sequencing were performed. The SNPsaurus and Novogene genome services (USA) were used. Paired‐end libraries were prepared using Nextera XT DNA Sample Preparation kit and Nextera XT Index kit (Illumina Inc. USA) according to the manufacturer's protocol. To find the closest genomes to each sequenced strain, whole genome sequence comparisons were performed using The Microbial Genomes Atlas (MiGA) webserver (http://microbial‐genomes.org/) (Rodriguez‐R et al. 2018).

### 2.4 Biodegradation of OWM in Flask Reactor

OWM was formulated in different ways: by mixing AW and OI (OI:2AW = OWM) or diluting the mix with one volume of tap water (OI:2AW:3H_2_O = OWM:H_2_O). Besides, for bacterial growth assays more diluted formulations (OWM, 4‐ and 10‐fold diluted in tap water) were used. The pH was regulated at 7.0 by using building lime. Then, for growth conditions under agitation, 150 μL of bacteria grown in LB for 16 h were resuspended in 15 mL of OWM or OWM:H_2_O and grown for 14 days with shaking at 180 rpm at 30°C. For static growth conditions, 1 mL of bacteria grown in LB for 16 h was resuspended in 14 mL of OWM:H_2_O and grown for 7 days statically at 30°C. Bacterial cells were enumerated by colony forming units (CFU) counted from serial dilutions in 150 mM NaCl plated onto LB agar plates. The samples were collected from the flasks to evaluate biodegradation efficiency, where 1 mL of starter culture grown in LB for 16 h was resuspended in 14 mL of medium formulated as described above.

### 2.5 Soluble Solids in Ether (SSE) Quantification

Quantification of SSE was performed as previously described with some adaptations (Baird et al. 2017). Briefly, 14 mL of OWM:H_2_O was inoculated with 1 mL of saturated culture of bacterial isolates grown in LB. After treatment, 10 mL were placed into a separatory funnel followed by the addition of 0.4 mL of concentrated hydrochloric acid, 2 mL of petroleum ether, and 6 mL of ethyl ether. The mixture was shaken vigorously for 2 min and left to rest for 2 h. Subsequently, 8 mL of distilled water was added, and the procedure was repeated. Once the phases had been separated, the aqueous layer was drained into the original sample container. The organic layer was drained through a funnel containing filter paper and 10 g Na_2_SO_4_ into a clean pre‐dried and pre‐weighed beaker. When a clear solvent layer was not obtained and an emulsion was observed, the emulsion and solvent layers were drained into a glass centrifuge tube and centrifuged for 5 min at approximately 2400 rpm to separate the phases. Then, the centrifuged material was transferred to a separatory funnel to restart the process. The aqueous layer and any remaining emulsions or solids were recombined in the separatory funnel to repeat the extraction twice more. Finally, the extracts were combined in the initial tared beaker. The solvent was evaporated, and the beaker was placed in a desiccator before weighing. The process was repeated until a constant weight was achieved.

### 2.6 Chemical Oxygen Demand (COD)

Chemical oxygen demand (COD) was measured with the HACH 8000 reactor digestion method, in a range of 200 to 15,000 mg L^−1^ (Hach), following protocols previously described by the manufacturer with the collaboration of Centro de Ingeniería Sanitaria (CIS) (Rosario, Santa Fe, Argentina). Briefly, 15 mL of waste mix was prepared and inoculated as described above. This parameter was evaluated after 7 days of treatment at 180 rpm at 30°C. Samples were analysed by biological triplicates. An experimental control was carried out with the addition of 1 mL of LB medium to the waste mix, as used for inoculations, maintained in similar conditions.

### 2.7 Phytotoxicity Assay

Hypocotyl elongation (HE) and root elongation (RE) were performed as previously described (Bagur‐González et al. 2011; Priac et al. 2017). Briefly, *Lactuca sativa* L. var. longifolia (lettuce) seeds were used. For each sample, 4 replicates of 20 seeds were placed into plastic containers with filter paper at the bottom as support. A final volume of 2 mL of samples treated for 7 days diluted 10‐fold with tap water was added. The containers were placed in the dark for 72 h at 25°C. Seedlings watered with tap water were used as a control. An additional experimental control was carried out with OWM:H_2_O without bacterial inoculation, maintained for 7 days under agitation at 30°C, as the inoculated samples, and used at similar conditions (10‐fold diluted). Finally, the length of the roots and hypocotyl was measured.

### 2.8 Sample Preparation for Nuclear Magnetic Resonance (NMR) Spectroscopy and ^1^H NMR Data Acquisition

Sample preparation was performed as described above. Briefly, after 7‐day treatment with shaking at 180 rpm at 30°C, samples were centrifuged 10 min at 13,000 rpm. The supernatant obtained was mixed with 600 μL of 0.2 M potassium phosphate buffer prepared in D_2_O containing 0.05% (w/v) of the internal standard 3‐(trimethylsilyl)propionic‐2,2,3,3‐d4 acid (TSP). The pH was adjusted to 3.1 with HCl and the mixtures were centrifuged at 13,500 rpm for 15 min at 4°C. A volume of 500 μL of the centrifuged solution was transferred into a 5‐mm NMR tube (Wilmad LabGlass) for ^1^H NMR analysis. ^1^H NMR spectra were recorded on a Bruker Avance III 700 MHz NMR spectrometer (Bruker Biospin, Rheinstetten, Germany) equipped with a 5‐mm TXI probe using a standard 1‐D noesy pulse sequence (noesygppr1d) with water presaturation. Proton spectra were acquired at 300 K. The mixing time was set to 10 milliseconds, the data acquisition period to 2.228 s, and the relaxation delay to 4 s. The ^1^H NMR spectra were acquired using 4 dummy scans and 32 scans with 64 K time domain points and a spectral window of 20 ppm. FIDs were multiplied by an exponential weighting function corresponding to a line broadening of 0.3 Hz. Proton spectra were referenced to the TSP signal (*δ* = 0 ppm) and analysed with TOPSPIN 3.6 (Bruker). Samples were run in triplicates and experiments were performed twice.

### 2.9 Residue Disposal Evaluation Through Phytotoxicity Assays

Onion bulbs (*Allium cepa* L. var. cepa) of the same size 50–60 mm, weighing about 90–100 g, were used following a protocol previously described (Firbas and Amon 2013; Bonciu et al. 2018) with some adaptations. Briefly, trays (without drain) containing 300 g of soil were irrigated with 150 mL of OWM:H_2_O after 7 days of bacterial treatment at 30°C with agitation at 180 rpm, as described above, or without inoculation treated in similar conditions. Then, dry soil kept for 7 days at room temperature was transferred to disposable cups, and the onion bulbs without roots were placed, watered with 50 mL of tap water, and maintained for 7 days until the length of roots was evaluated.

On the other hand, disposable cups containing 120 g of soil were prepared and lettuce seeds were planted. After that, they were irrigated with 2.5 mL of waste mix treated for 7 days, as described above, diluted 20‐fold with tap water. In parallel, the same volume of tap water and 2.5 mL of waste mixed without treatment with the same dilution factor applied were used as experimental controls. The seeded and watered cups were taken in a growth chamber in incandescent light at 28°C with a photo period of 16 h. Finally, the evaluation of fresh and dry weight was carried out in plants after 21 days from sowing.

### 2.10 Thin Layer Chromatography (TLC)

Samples (untreated OWM:H_2_O and waste mix treated with *Cytobacillus* sp. OWB‐43) were loaded (100 μg/spot unless otherwise stated) onto Aluminium TLC sheets coated with silica gel 60 F254 (Merck, Germany). Sample preparation was performed as described above and after treatment for 7 days with shaking at 180 rpm at 30°C, samples were centrifuged 10 min at 13,000 rpm and 30 μL of each one was subjected to chromatographic separation. The samples were applied in bands of 4 mm drying with air spray gas. The TLC plates were developed with different solutions (chloroform: ethyl acetate: acetic acid (10:10:1), methanol: acetic acid (19:1), methanol: water: acetic acid (10:10:1), absolute ethanol and ethanol: water (85:15)) at 25°C in a glass chamber (Bueloni, Rosario, Argentina), to a migration distance of 65 mm. After development, the plates were dried and images were captured under white light, UV 302 nm, UV 365 nm, and UV 254 nm. At 254 nm, aromatic or highly conjugated molecules absorb. At 302 nm, other compounds may absorb, and at 365 nm, compounds with natural fluorescence can be detected. Non‐fluorescent compounds may remain invisible at 365 nm unless they absorb UV light, in which case they can appear as dark spots (Nichols 2021).

### 2.11 Bioautography Assay

For bioautographic phytogrowth‐inhibitory bioassay (BPIB), preparative TLC plates were developed and once the solvent was completely removed, the plates were subjected to BPIB. The protocol was adapted from bibliography (Inoue et al. 1992; Arellanes et al. 1996). Briefly, the plate was evaluated under UV detection (302, 365, and 254 nm), and different regions were carefully cut. Subsequently, the plate regions were put into a Petri dish, coated with 0.5% (w/v) agar, and phytotoxicity tests were carried out. For this purpose, 15 lettuce seeds were sown and incubated for 72 h. Finally, seedling development was evaluated by measuring hypocotyl elongation (HE) and root elongation (RE). A TLC plate performed under the same running conditions (without seeding samples) was used as control. The experiment was repeated three times.

### 2.12 Statistical Analysis

The results were expressed as the mean values with standard deviations (SD) in bar graphs or as the median values in box plots. Statistical significance was determined by ANOVA when assumptions of normality (Shapiro–Wilk's test) and homogeneity of variance (Bartlett's test) were met. When differences were observed by ANOVA, the Tukey's test was applied to compare all data groups. When the assumptions of normality and homogeneity of variance were not met, Kruskal‐Wallis's test and Dunn's test to compare all data groups were used.

## 3 Results

### 3.1 Isolation and Characterisation of Bacteria From Olein Industry Waste

A search for bacterial isolates was performed on different industrial waste samples and a total of 50 bacterial strains were obtained, in which 10 were isolated from AW, 30 from OI and 10 from the enrichment strategy. Different morphological isolates were systematically re‐isolated until pure cultures were achieved. The isolated pure strains were named starting with the letters OWB for “olein waste bacteria”, followed by consecutive numbers. After Gram staining, we found that 80% were Gram positive bacteria and 20% were Gram negative bacteria (data not shown). Identification by 16 s rRNA amplification or biotyping MALDI‐TOF assay revealed the presence of *Bacillus subtilis* OWB‐10 and *Bacillus velezensis* OWB‐30 isolated from AW and OI, respectively, whereas *Pseudomonas aeruginosa* OWB‐41, *Lysinibacillus* sp. OWB‐42, *Cytobacillus* sp. OWB‐43, *Enterobacter* sp. OWB‐48 and *Niallia* sp. OWB‐49 were obtained after the enrichment strategy (Table 1). *Bacillus*, *Pseudomonas*, and *Cytobacillus* are prominent bacterial genera widely utilised in environmental biotechnology for their distinct metabolic pathways (Sarwar and Kremer 1995) and ability to break down xenobiotic compounds and heavy metals (Arora 2020), organic pollutants (Hu et al. 2023), and hydrocarbons (Alim et al. 2025).

**TABLE 1:** Identification of strains isolated and characterised in this work.

Isolate	Identification method					
	MALDI TOF		16S rRNA sequencing		Whole genome sequencing (WGS)	Identification
	Organism (best match)	Score value	Organism (best match)	Similarity (%)		
OWB‐10	ND	ND	*Bacillus tequilensis* KCTC 13622	99.93	*Bacillus subtilis* Accession number: JAXUFM000000000	*Bacillus subtilis* OWB‐10
OWB‐30	ND	ND	*Bacillus siamensis* KCTC 13613	99.00	*Bacillus velezensis* Accession number: JAXUFL000000000	*Bacillus velezensis* OWB‐30
OWB‐41	*Pseudomonas aeruginosa*	2.36	*Pseudomonas aeruginosa* strain JCM 5962	100.00	*Pseudomonas aeruginosa* Accession number: JAXUFK000000000	*Pseudomonas aeruginosa* OWB‐41
OWB‐42	*Lysinibacillus xilaniliticus/boronitolerans*	2.20	*Lysinibacillus boronitolerans* T‐10a	99.71	ND	*Lysinibacillus* sp. OWB‐42
OWB‐43	*Bacillus megaterium*	1.57	*Cytobacillus kochii* WCC 4582	100.00	*Cytobacillus* sp. Accession number: JAYEFF000000000	*Cytobacillus* sp. OWB‐43
OWB‐48	ND	ND	*Enterobacter hormaechei* subsp. *Xiangfangensis* LMG 27195	99.71	ND	*Enterobacter* sp. OWB‐48
OWB‐49	ND	ND	*Niallia circulans* ATCC 4513	99.72	ND	*Niallia* sp. OWB‐49

Abbreviation: ND: not determined.

*B. subtilis* OWB‐10 and *B. velezensis* OWB‐30 tested positive for all enzymatic activities examined and surfactant production, while the other strains showed varied results. *P. aeruginosa* OWB‐41 was negative only for cellulase activity. *Cytobacillus* sp. OWB‐43 was positive for lipase and lecithinase activities but negative for the other activities tested. *Lysinibacillus* sp. OWB‐42, *Enterobacter* sp. OWB‐48 and *Niallia* sp. OWB‐49 were positive only for lipase activity. Among these, *Lysinibacillus* sp. OWB‐42 and *Cytobacillus* sp. OWB‐43 showed the highest lipase activity, as evidenced by the formation of a larger precipitates of calcium salts around the macrocolony (Figure 1). Moreover, to further characterise the microbial species, we cultured them and generated growth curves, from which the growth rate was calculated. All strains showed good performance to grow in a commercial media like LB under controlled conditions (Figure S3).

**FIGURE 1:**
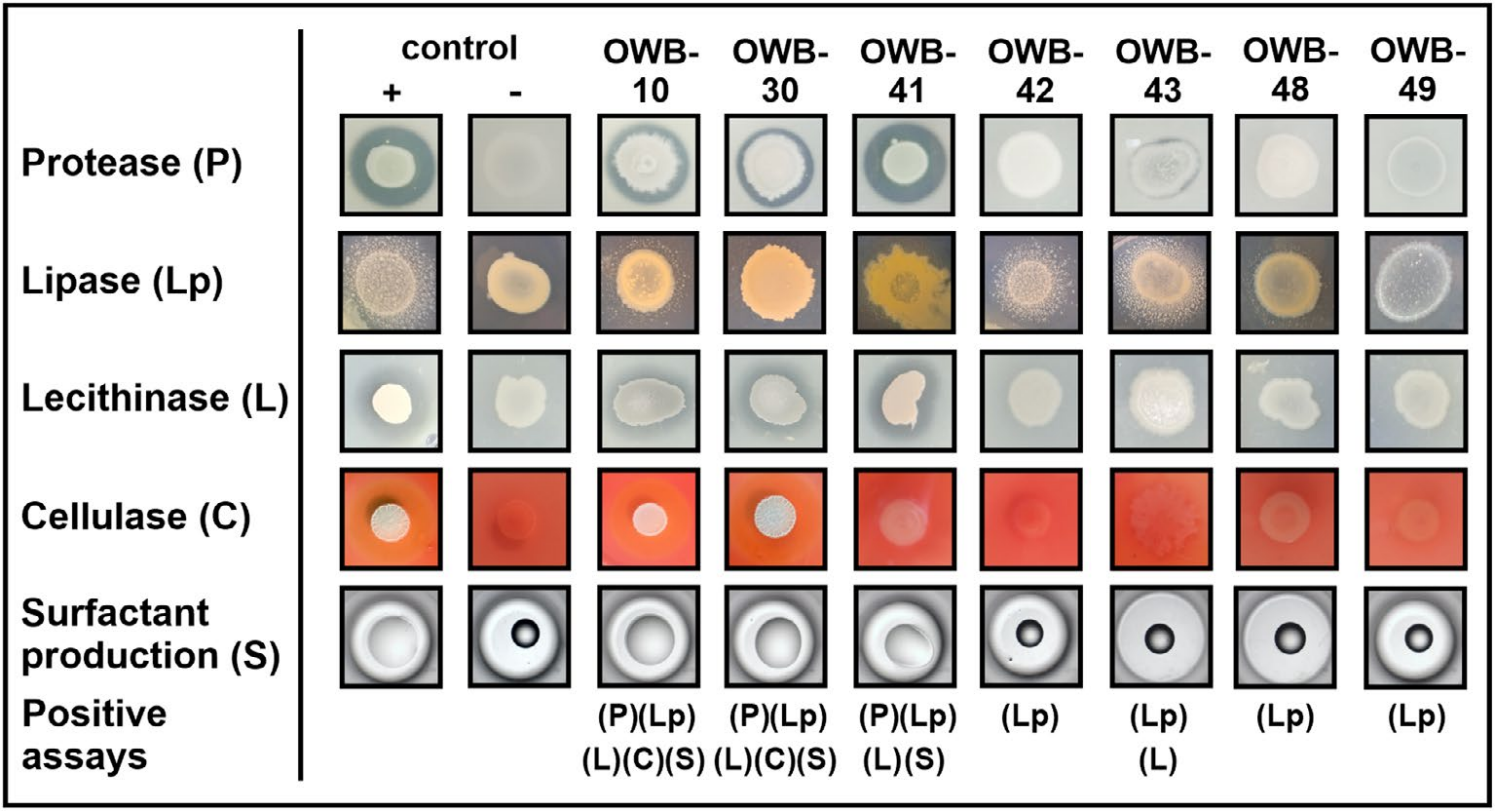
Enzymatic activities and surfactant production. Protease (P), Lipase (Lp), Lecithinase (L), Cellulase (C) activities and Surfactant production (S) were tested for *B. subtilis* OWB‐10 (OWB‐10), *B. velezensis* OWB‐30 (OWB‐30), *P. aeruginosa* OWB‐41 (OWB‐41), *Lysinibacillus* sp. OWB‐42 (OWB‐42), *Cytobacillus* sp. OWB‐43 (OWB‐43), *Enterobacter* sp. OWB‐48 (OWB‐48), and *Niallia* sp. OWB‐49 (OWB‐49). Representative images of each assay are shown. At the bottom, letters indicate which strains tested positive for each activity. *P. aeruginosa* PAO1 was used as a positive control for protease, lipase, lecithinase activities and surfactant production, while *B. subtilis* 168 served as a positive control for cellulase activity. *E. coli* BL21(DE3) was included as a negative control.

Due to the promising activities of *B. subtilis* OWB‐10, *B. velezensis* OWB‐30, *P. aeruginosa* OWB‐41, and *Cytobacillus* sp. OWB‐43, we sequenced their genomes (https://www.ncbi.nlm.nih.gov/bioproject/1053253). Final genome assembly data are provided in the Supporting Information (Table S1 and Figure S4).

### 3.2 Bacterial Growth

To analyse whether the selected isolates could use the residual mixture as a nutrient source, bacterial growth was evaluated under agitation conditions in OWM and OWM:H_2_O. Bacterial strains grown ON in LB were used to inoculate OWM and OWM:H_2_O. *P. aeruginosa* OWB‐41 was the only isolate capable of growing in OWM, increasing from 10^7^ CFU mL^−1^ at time 0 to 3 × 10^10^ CFU mL^−1^ after 3 days and remained at this concentration until day 15 (Figure 2A). Conversely, all isolates were able to grow in OWM:H_2_O (Figure 2B). The highest growth was observed for *P. aeruginosa* OWB‐41 and *Enterobacter* sp. OWB‐48, which reached 2.6 × 10^10^ CFU mL^−1^ and 1.2 × 10^10^ CFU mL^−1^, respectively, after 7 days. At the same timepoint, *Cytobacillus* sp. OWB‐43 reached 3.7 × 10^8^ CFU mL^−1^ and *Niallia* sp. OWB‐49 1.8 × 10^8^ CFU mL^−1^. Both *B. subtilis* OWB‐10 and *B. velezensis* OWB‐30 showed similar growth after 7 days, reaching 1.9 × 10^8^ CFU mL^−1^ and 1.8 × 10^8^ CFU mL^−1^, respectively. *Lysinibacillus* sp. OWB‐42 reached 2.7 × 10^8^ CFU mL^−1^ (Figure 2B). Therefore, all strains were able to use the waste mix as a nutrient source when diluted with tap water. Besides, growth was evaluated under similar conditions of agitation and temperature in OWM with 4‐ and 10‐fold dilution of tap water. However, no differences were observed in the maximum population reached compared to 1‐fold dilution (data not shown). Therefore, the 1‐fold diluted OWM was chosen for further analysis.

**FIGURE 2:**
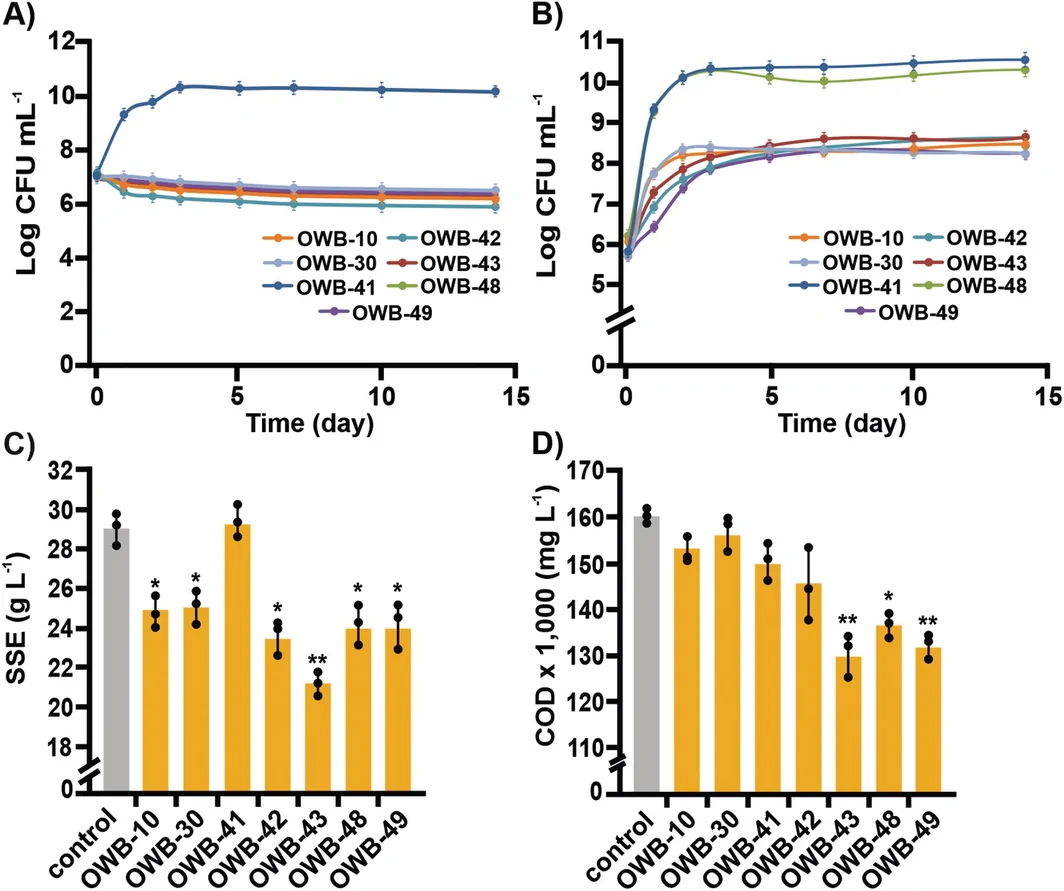
Bacterial growth and olein residual treatment. Growth of *B. subtilis* OWB‐10 (OWB‐10), *B. velezensis* OWB‐30 (OWB‐30), *P. aeruginosa* OWB‐41 (OWB‐41), *Lysinibacillus* sp. OWB‐42 (OWB‐42), *Cytobacillus* sp. OWB‐43 (OWB‐43), *Enterobacter* sp. OWB‐48 (OWB‐48), and *Niallia* sp. OWB‐49 (OWB‐49) was evaluated in (A) conditioned olein waste mix without dilution (OWM) and (B) diluted by half in tap water (OWM:H_2_O). (C) Quantification of remnant soluble solids in ether (SSE) (g L^−1^) and (D) chemical oxygen demand (COD) (g L^−1^) after 7 days of treatment under agitation conditions at 30°C. Values represent the means of three samples and are representative of three independent experiments. Error bars are standard deviations. Conditions that presented statistically significant differences are indicated with asterisks: *p < 0.05, **p < 0.01.

### 3.3 Physicochemical Evaluation of OWM:H_2_O

Total fats, expressed as soluble solids in ether (SSE), showed a value of 29 g L^−1^, chemical oxygen demand (COD) was 160 g L^−1^, and total solids were 96 g L^−1^. Electrical conductivity (EC) measured 13.5 dS m^−1^. In addition, sulfates, nitrates, nitrites, and chlorides, and some elements such as Fe, Zn, Na, K, Mg, Ca, Al, and P were measured (Table S2).

### 3.4 *Cytobacillus* sp. OWB‐43 Reduces SSE and COD of Conditioned Residual Mixture

To quantify the capability of the isolates to transform the waste treatment residues, soluble solids in ether (SSE) and chemical oxygen demand (COD) were measured. Based on growth curves and with the idea of using the minimum amount of water to dilute samples, OWM:H_2_O was used for bacterial treatments. *Cytobacillus* sp. OWB‐43 was the most effective strain in reducing SSE, achieving a 24.80% reduction after 7 days of treatment compared to the control (Figure 2C). Notably, all tested strains except *P. aeruginosa* OWB‐41 demonstrated the ability to reduce SSE. Regarding COD, the most efficient strains after 7 days of treatment were *Cytobacillus* sp. OWB‐43 and *Niallia* sp. OWB‐49, which reduced COD by 18.75% and 17.71%, respectively, compared to the control. *Enterobacter* sp. OWB‐48 reduced COD by 14.58%. However, *B. subtilis* OWB‐10, *Bacillus* sp. OWB‐30, *P. aeruginosa* OWB‐41, and *Lysinibacillus* sp. OWB‐42 did not improve upon control values (Figure 2D).

### 3.5 *Cytobacillus* sp. OWB‐43 Reduces Phytotoxicity of Conditioned Residual Mixture

Phytotoxicity tests, among them HE and RE quantifications using *Lactuca sativa* L. var. longifolia, are widely used due to their simplicity, low cost, and high sensitivity to trace levels of toxic substances. These tests are recommended as bioindicators to determine the toxicity of soil and water samples (Priac et al. 2017). In this study, lettuce seedlings irrigated with OWM:H_2_O treated with *Cytobacillus* sp. OWB‐43 and *Niallia* sp. OWB‐49 showed significantly improved HE compared to seedlings watered with untreated waste mix, used as a control (Figure 3A,B). Specifically, medians hypocotyl length were 9.02 mm (*p‐value* 1 × 10^−8^) and 9.04 mm (*p‐value* 1 × 10^−8^) for *Cytobacillus* sp. OWB‐43 and *Niallia* sp. OWB‐49, respectively, differently from the untreated control (3.46 mm) (Figure 3A,B). For RE, significant improvements were observed with *Cytobacillus* sp. OWB‐43 or *B. subtilis* OWB‐10. The medians were 10.71 mm (*p‐value* 1 × 10^−8^) and 8.10 mm (*p‐value* 0.023), respectively, compared to 5.88 mm in the untreated control (Figure 3C). No significant differences were observed for other tested bacterial strains. As an additional control, seedlings irrigated with tap water showed the highest growth showing a median for hypocotyl and root length of 10.44 and 14.57 mm, respectively.

**FIGURE 3:**
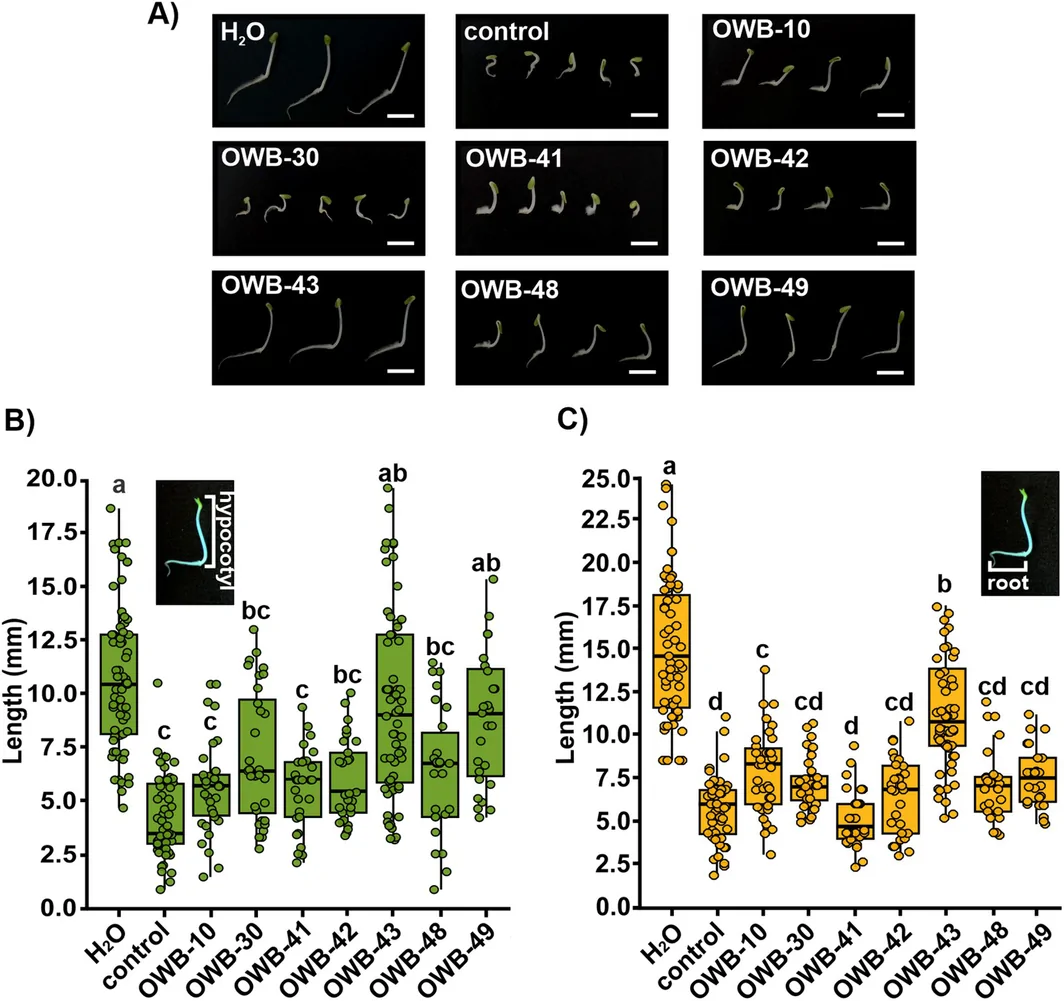
Phytotoxicity assay. *Lactuca sativa* L. (lettuce) seeds were placed into plastic containers with filter paper as support. Seedlings were watered with 2 mL of samples treated for 7 days under agitation conditions at 30°C with *B. subtilis* OWB‐10 (OWB‐10), *B. velezensis* OWB‐30 (OWB‐30), *P. aeruginosa* OWB‐41 (OWB‐41), *Lysinibacillus* sp. OWB‐42 (OWB‐42), *Cytobacillus* sp. OWB‐43 (OWB‐43), *Enterobacter* sp. OWB‐48 (OWB‐48), and *Niallia* sp. OWB‐49 (OWB‐49). All samples were diluted 10‐fold. Tap water (H_2_O) was used as a control, and an additional experimental control was carried out with OWM:H_2_O supplemented with 1 mL of LB medium under similar treatment conditions (control). (A) Representative images of seedlings, (B) hypocotyl length (mm), and (C) root length (mm) after incubation in the dark for 72 h at 25°C. Scale bar = 1 cm. Letters are assigned to groups with statistically similar means, and groups with different letters are significantly different.

Next, to compare the chemical composition of the untreated sample and the OWM:H_2_O treated with *Cytobacillus* sp. OWB‐43, NMR was performed (Figure 4A). Despite the expected complexity, our analysis revealed interesting differences. Some of the signals that are present in the untreated samples disappeared after treatment and were assigned to propionate, acetate, levulinate (Figure 4B), and glycerol (Figure 4C). Additionally, other signals, such as at 1.65 and 2.60 ppm, showed significant variations between the control and the OWB‐43‐treated sample, although their identities could not be assigned.

**FIGURE 4:**
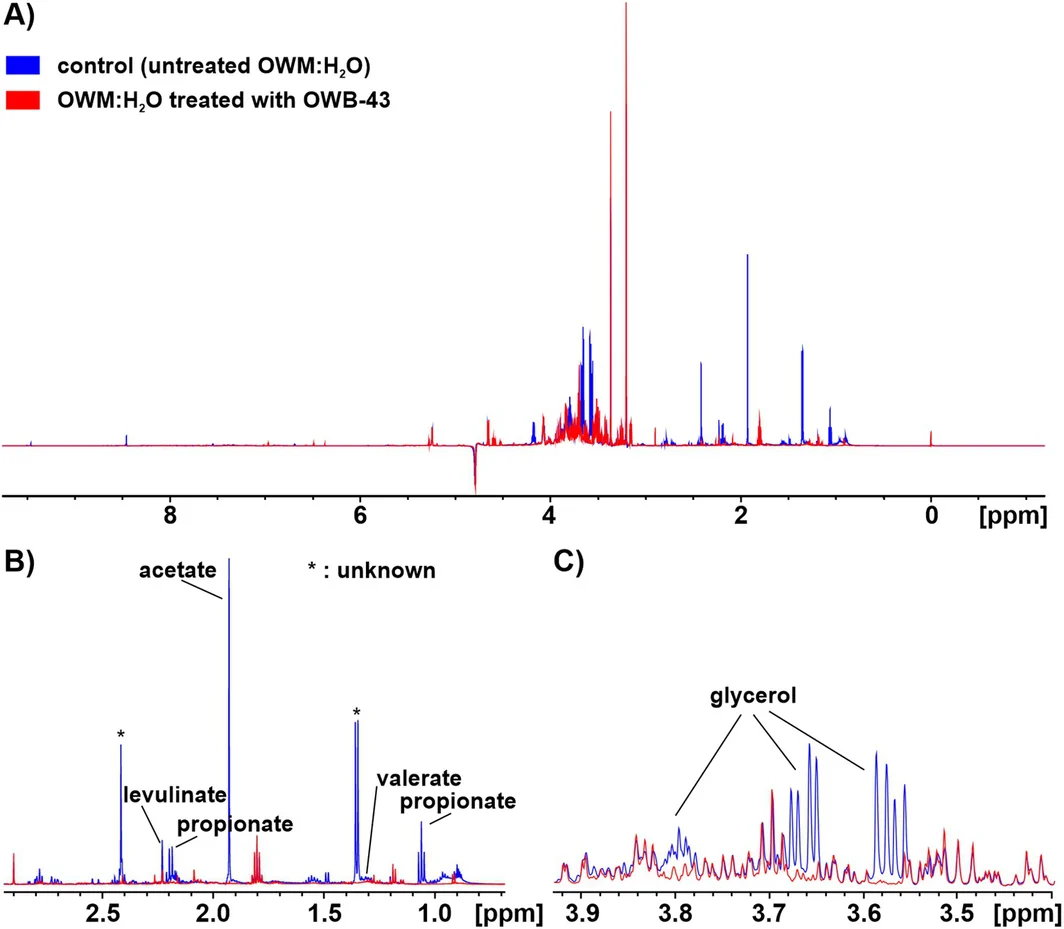
Comparison between the ^1^H spectra obtained by NMR for the untreated OWM:H_2_O (blue) and OWM:H_2_O treated with *Cytobacillus* sp. OWB‐43 (red). (A) Total spectrum obtained in the range of 0 to 9 ppm. (B) Spectral amplification in the range between 0.7 to 2.9 ppm and (C) 3.40 to 3.91 ppm. The scale used was adjusted to the intensities of the peaks obtained for each region.

### 3.6 *Cytobacillus* sp. OWB‐43 Treatment Enables Safe Disposal of Residual Waste in Soil

*Cytobacillus* sp. OWB‐43 was the strain selected to perform the final waste treatment due to its high effectiveness to reduce COD, SSE, and phytotoxicity. Therefore, to evaluate whether the treated waste mixture could be safely disposed of on soil, thus completing the bioremediation process through landfarming, its effect was assayed.

Onion bulbs from soil watered with tap water developed roots with a median length of 52.71 mm after 7 days. Bulbs watered with waste mix treated with *Cytobacillus* sp. OWB‐43 developed roots with a median length of 26.43 mm. Interestingly, bulbs irrigated with untreated OWM:H_2_O did not develop roots (Figure 5A,B), indicating residue high phytotoxicity to be disposed on soil.

**FIGURE 5:**
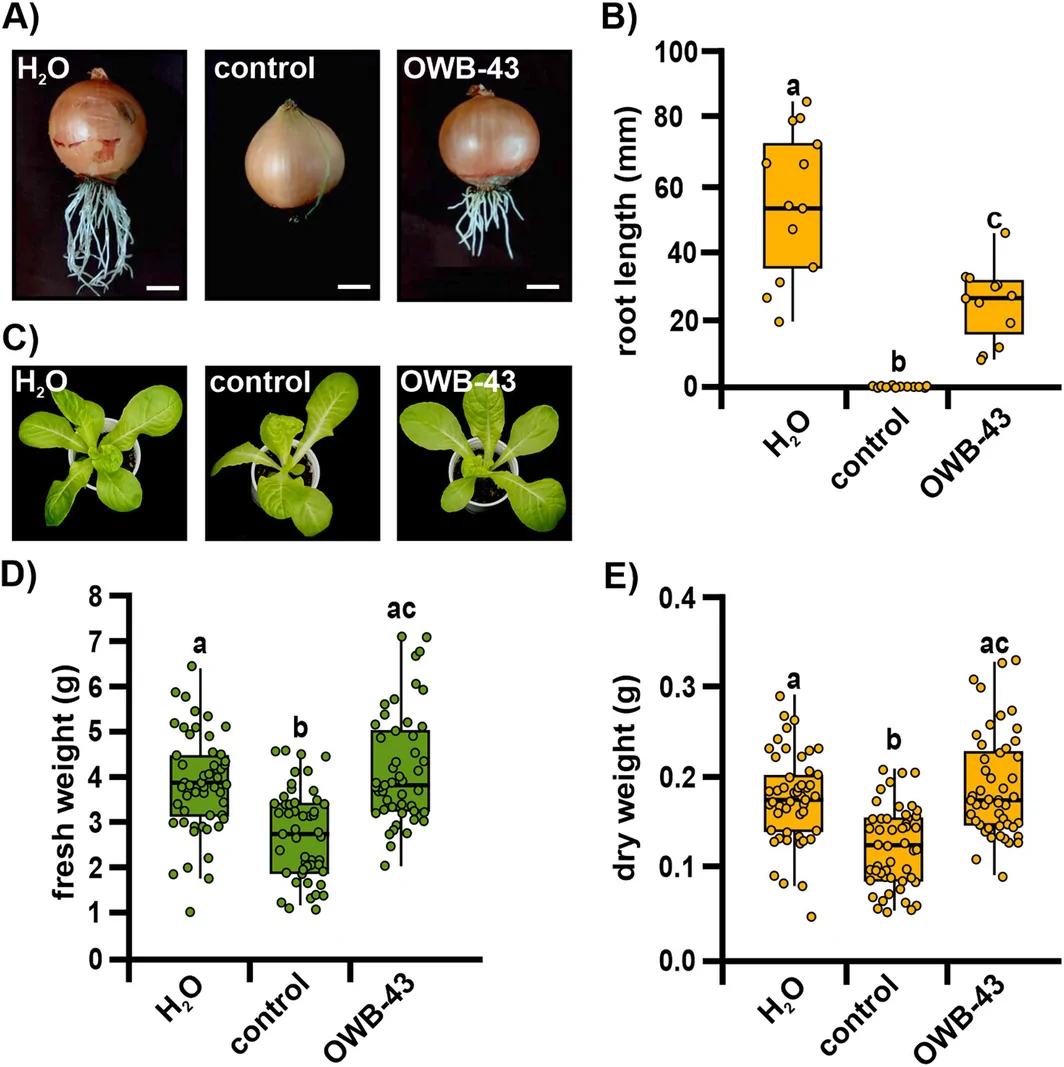
Final disposal evaluation. Containers with 300 g of soil were irrigated with 150 mL of: Tap water (H_2_O), OWM: H_2_O plus 1 mL of LB medium maintained under similar conditions (control), or waste mix treated with *Cytobacillus* sp. OWB‐43 for 7 days (OWB‐43). After 7 days of irrigation, the dry soil was transferred to disposable cups and onion bulbs (*Allium cepa* L.) without roots were placed into the soil, watered with 50 mL of tap water, and maintained for 7 days before root length evaluation. (A) Representative images and (B) root length (mm) quantification. Lettuce seeds (*Lactuca sativa* L.) were planted in disposable cups containing 120 g of soil and irrigated with 2.5 mL of tap water (H_2_O), OWM:H_2_O plus 1 mL of LB medium (control), or 20‐fold diluted waste mix treated with *Cytobacillus* sp. OWB‐43 for 7 days (OWB‐43). The cups were placed in a plant culture chamber. (C) Representative images, and quantification of (D) fresh and (E) dry weight of plants after 21 days of sowing are shown. Scale bar = 1 cm. Letters are assigned to groups with statistically similar means, and groups with different letters are significantly different.

Lettuce plants grown for 21 days were also evaluated. Given lettuce's sensitivity to high salinity—and, by extension, to electrical conductivity, which was found to be elevated in OWM:H_2_O (Table S2), even after bacterial treatment (Figure S5)—a 20‐fold dilution was applied before use. Interestingly, no statistically significant differences were observed in the aerial parts of lettuce plants irrigated with tap water compared to the ones irrigated with waste mix pre‐treated with *Cytobacillus* sp. OWB‐43 (Figure 5C–E). At 21 days post‐sowing, fresh and dry weight of plants irrigated with treated OWM:H_2_O were 3.80 and 0.17 g, respectively, nearly identical to the control values, 3.83 and 0.17 g. Nevertheless, statistically significant differences were observed between plants irrigated with untreated OWM:H_2_O (2.73 and 0.12 g of fresh and dry weight, respectively) and the same waste mix treated with *Cytobacillus* sp. OWB‐43. An increase of 28.00% in fresh weight and 27.90% in dry weight was observed in plants irrigated with treated residue (Figure 5D,E).

### 3.7 Several Compounds May Contribute to the Phytotoxicity of the Waste

To separate and analyse components in the complex OWM:H_2_O mixture after treatment, thin layer chromatography was performed (Figure S6). Among the MPs tested—chloroform:ethyl acetate:acetic acid (10:10:1), methanol:acetic acid (19:1), methanol:water:acetic acid (10:10:1), absolute ethanol, and ethanol:water (85:15) (Figure S6A–SE, respectively)—absolute ethanol showed distinct separation patterns between untreated and treated samples and was therefore selected for further characterisation. Preparative TLC plates with this MP were divided into five zones to identify regions responsible for phytotoxicity. This was performed using the bioautographic phytogrowth‐inhibitory bioassay (BPIB) (Figure S7A). Only region 1, corresponding to the application spot, showed significant differences in HE and RE between untreated and treated samples (Table S3). In untreated OWM:H_2_O, HE and RE were 6.0 and 5.8 mm, respectively, while bacterial treatment improved the values to 8.4 mm for HE and 10.3 mm for RE (Table S3 and Figure S7B).

To improve compound separation and enhance migration beyond the application spot, a more polar solvent system (ethanol: water 85:15) was used as the MP (Figure S6E). This allowed the detection of additional toxic compounds in previously unresolved TLC regions. BPIB analysis with this system revealed that both HE and RE were significantly improved after bacterial treatment compared to the untreated sample (Figure 6A,B, respectively), with significant differences in regions 2, 3, 4, and 5 (Table 2). Specifically, in region 2: RE increased from 6.7 mm (untreated) to 10.8 mm (treated); in region 3: RE increased from 5.6 to 12.0 mm; in region 4: HE increased from 5.3 to 9.8 mm; and in region 5: RE increased from 5.4 mm to 10.6 mm (Figure 6 and Table 2). Strikingly, region 4 also showed the highest RE values after treatment, indicating a positive effect (Figure 6B and Table 2). These results suggest that several compounds contribute to the overall phytotoxic effect of the untreated sample, and that *Cytobacillus* sp. OWB‐43 treatment effectively reduces this toxicity across various compound classes.

**FIGURE 6:**
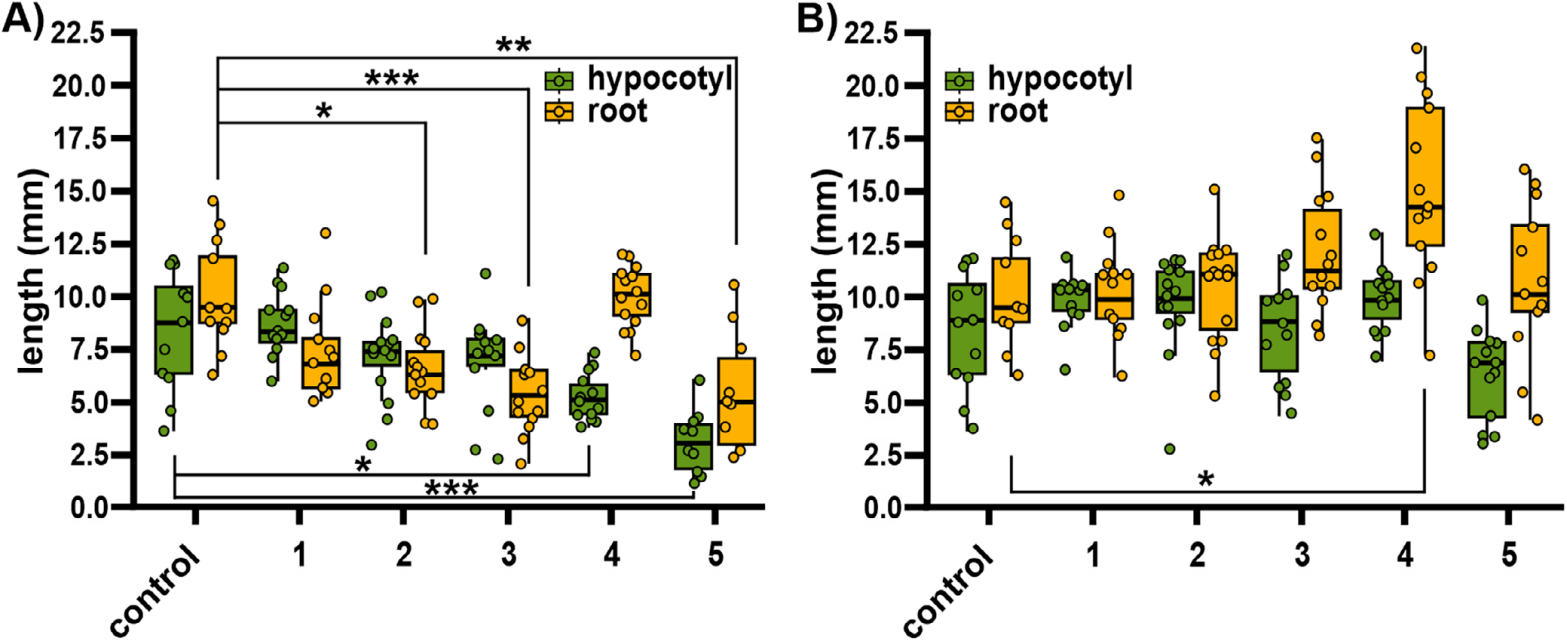
Bioautographic phytogrowth‐inhibitory bioassay (BPIB). Box plots corresponding to the hypocotyl and root length of the seedlings obtained onto TLC plate after incubation for each treatment and region. BPIB using ethanol:Water (85:15) as mobile phase for chromatographic separation was performed for (A) the untreated OWM and (B) OWM inoculated with *Cytobacillus* sp. OWB‐43. As experimental control, TLC maintained under the same conditions without sample seeding was evaluated. The corresponding statistical analyses were performed. Conditions that presented statistically significant differences are indicated with asterisks: *p < 0.05, **p < 0.01, ***p < 0.001.

**TABLE 2:** Average values of hypocotyl and root length from TLC regions evaluated by the autobiography assay, for the untreated sample (control) or bacterial treatment with *Cytobacillus* sp. OWB‐43 (OWB‐43).

Region	Hypocotyl length (mm)		*p‐value*	Root length (mm)		*p‐value*
	Control	OWB‐43		Control	OWB‐43	
1	8.7	9.9	1.00	7.3	10.1	0.50
2	7.0	10.1	0.09	6.7	10.8	0.02*
3	7.1	8.5	1.00	5.6	12.0	0.00003***
4	5.3	9.8	0.00005***	10.1	15.8	0.30
5	3.1	6.3	0.90	5.4	10.6	0.03*

*Note:* Conditions that showed statistically significant differences are indicated with asterisks: *p < 0.05, **p < 0.01, ***p < 0.001. Mobile phase: Ethanol: Water (85:15).

### 3.8 Static Growth of *Cytobacillus* sp. OWB‐43 Also Leads to Phytotoxicity Reduction

In order to assess the feasibility of waste treatment without the energy input required for agitation, we evaluated whether *Cytobacillus* sp. OWB‐43 could grow under static conditions. The strain successfully grew, reaching a population of 1.5 × 10^9^ CFU mL^−1^ after 7 days (Figure 7A), approximately 100‐fold increase from the initial inoculum.

**FIGURE 7:**
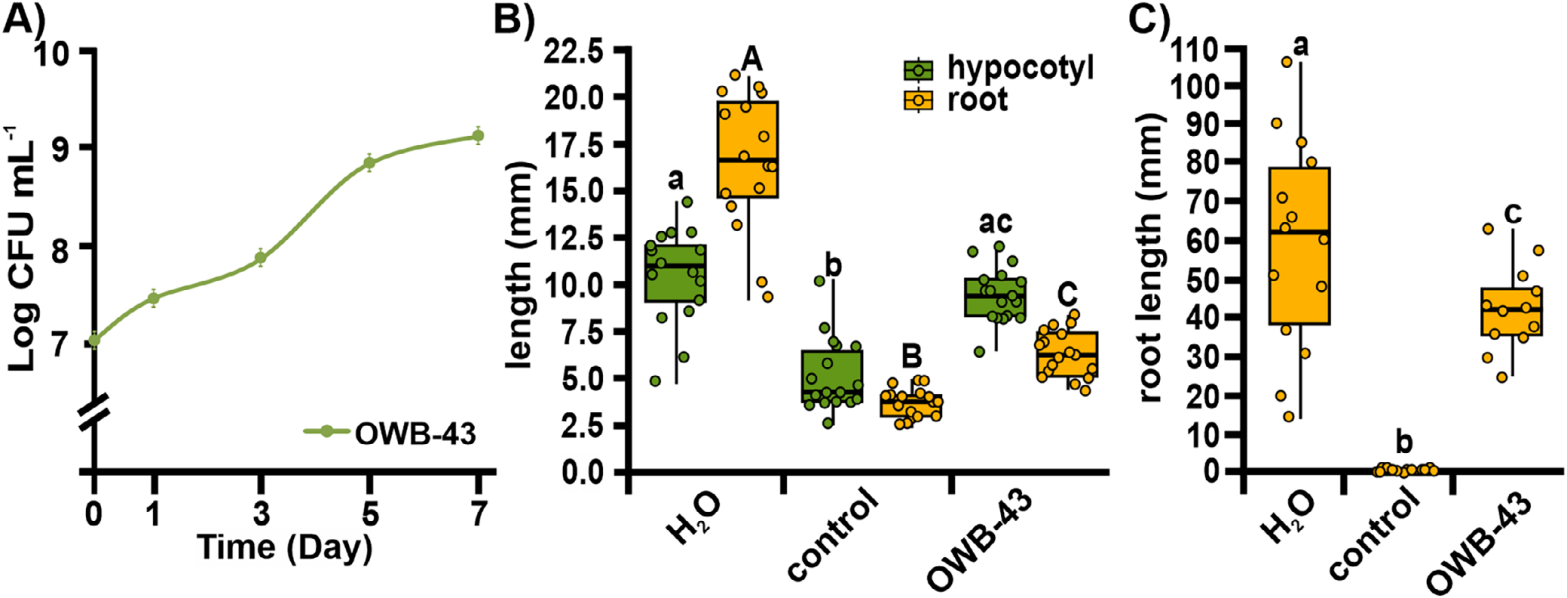
Static treatment. (A) *Cytobacillus* sp. OWB‐43 (OWB‐43) growth under static conditions at 30°C. (B) Hypocotyl and root length (mm) of lettuce seedlings irrigated with waste mix treated for 7 days with *Cytobacillus* sp. OWB‐43 (OWB‐43) under static conditions. (C) Root length (mm) of onion bulbs used as biosensors watered with tap water (H_2_O), untreated OWM:H_2_O plus 1 mL of LB medium (control) and waste mix treated with OWB‐43 for 7 days under static incubation. Letters are assigned to groups with statistically similar means, and groups with different letters are significantly different.

Lettuce seedlings watered with OWM:H_2_O previously treated with *Cytobacillus* sp. OWB‐43 for 7 days in static conditions showed a median HE of 9.3 mm, compared to 4.3 mm with untreated OWM:H_2_O, and 11.1 mm with tap water. For RE, the bacterial‐treated OWM:H_2_O group reached 6.2 mm, while this parameter was 3.8 mm in the untreated OWM:H_2_O control, and 16.6 mm for water control (Figure 7B). When onion bulbs were used, a similar behaviour was observed. Root length values were 61.9 mm for water, 41.5 mm for onions irrigated with treated OWM:H_2_O and no root development was observed when untreated OWM:H_2_O was used (Figure 7C).

## 4 Discussion

Edible oil industries generate by‐products such as oleins, which have multiple applications including its use as a feedstock for biodiesel production and as an ingredient in animal feed. However, the extraction of oleins also generates AW and OI residues (Rajkumar et al. 2010; Gharby 2022).

Although AW and OI are classified as non‐toxic and non‐hazardous, they exhibit low pH due to the acidulation process and have a high organic load. The accumulation of protons (H^+^) in highly acidic environments alters the electrical charge of biological molecules, thereby affecting their structure and function. In addition, weak organic acids can diffuse across the lipid membrane and dissociate within the cell, releasing protons into the cytoplasm. This intracellular acidification disrupts pH homeostasis and collapses the proton motive force across the membrane (Lund et al. 2020). These features likely contributed to the high proportion of Gram‐positive bacteria found in these environments. Specifically, Firmicutes, such as *Bacillus* spp., are known to form spores that can resist the acidic conditions commonly found in industrial waste (Bressuire‐Isoard et al. 2018). Particularly, *B. subtilis* maintains a stable cytoplasmic pH between 7.3 and 7.6 during vegetative growth in a range of environmental pH between 6.0–9.0 (Wilks et al. 2009). Additionally, low pH is known to prevent induced spore germination in *B. subtilis*, *cereus*, and *megaterium* (Wuytack and Michiels 2001). Interestingly, open‐air pools of untreated waste with unadjusted pH remain unchanged for years, underscoring the inhibitory nature of this environment and the lack of microbial activity under these conditions. Indeed, no growth was detected when non‐conditioned residual mix was inoculated with the isolated strains, both under agitation conditions and on agar plates (Figure S8A), while adjusted pH to 7 allowed growth (Figure S8B). In many environments, prolonged exposure to contaminants can lead to the emergence of specialised microbial communities capable of degrading those compounds (Potts et al. 2022). However, in our case, the lack of microbial activity in the untreated waste, likely due to its low pH and high organic load, suggests that such adaptive evolutionary processes have not occurred. This makes the isolation of effective degrading strains from the waste environment itself unlikely.

In this study, we identified bacterial isolates with promising enzymatic activities. Among them, *B. subtilis* OWB‐10 and *Bacillus* sp. OWB‐30. Correlation between bacterial enzymatic activity and bioremediation potential has been previously reported, in which microorganisms play a crucial role in degradation or transformation of pollutants into less toxic substances (Narayanan et al. 2023). For example, microbial communities in long‐term petroleum‐contaminated soil showed polycyclic aromatic hydrocarbons (PAHs) degradation and enzymatic activity (Markowicz et al. 2016). All isolated strains were able to grow under water‐diluted conditions. Although bacterial growth was observed even at higher dilution levels, we selected a 50% dilution to minimise water use. Differences in growth performance were observed at different OWM concentrations; however, COD, SSE, and phytotoxicity were not measured comparatively at different waste mixture conditions. *B. subtilis* OWB‐10 and *Bacillus* sp. OWB‐30 did not demonstrate the best performance in the conditioned waste mix, or reductions in COD and phytotoxicity. Instead, *P. aeruginosa* OWB‐41 and *Enterobacter* sp. OWB‐48 showed better growth in the waste, with *P. aeruginosa* OWB‐41 growing even without water dilution, possibly due to more efficient lipases, though this hypothesis requires further validation. *P. aeruginosa* OWB‐41 was not able to reduce SSE, COD and phytotoxicity, while *Enterobacter* sp. OWB‐48 showed decrease in SSE and COD. Among the isolates evaluated, *Cytobacillus* sp. OWB‐43 was selected as the most promising candidate for further bioremediation studies. This strain exhibited both lipase and lecithinase activities, showed robust growth in the conditioned OWM:H_2_O, and effectively reduced SSE and COD parameters. In addition, treatment with OWB‐43 decreased the phytotoxicity of the waste, as evidenced by improved lettuce growth in the treated samples. The COD of untreated waste used in this work (160,200 ± 5500 mg L^−1^) was almost six‐fold higher than those reported for similar effluents, such as from a vegetable oil refinery in India where the COD of the processed wastewater was 29,120 ± 4500 mg L^−1^ (Pandey et al. 2003). Bacterial treatment of water produced after gas‐to‐liquid process achieved 60% COD reduction after 7 days of incubation with *Alcaligenes faecalis*, *Stenotrophomonas* sp., and *Ochrobactrum* sp. corresponded to approximately 4200 mg L^−1^ (Surkatti et al. 2021). In this study, treatment with *Cytobacillus* sp. OWB‐43 achieved a COD reduction of 18.75% (~30,000 mg L^−1^) after 7 days, outperformed previous reports (Surkatti et al. 2021). Generally, wastewater with COD values below 1000 mg L^−1^ is aerobically treated, whereas anaerobic treatment is recommended for COD values above 4000 mg L^−1^ (Chan et al. 2009). In our case, the implementation of anaerobic treatment was limited by the high sulfate content (9.2 g L^−1^), which could inhibit anaerobic microbial activity. Nonetheless, aerobic treatment had proven effective even at high COD levels (75,900 mg L^−1^). For instance, an effluent from palm oil was successfully treated with *B. cereus* and *B. subtilis*, achieving a 90% COD reduction within 5 days (Bala et al. 2015). A substantial difference with our study was that the starting material, particularly in Total Suspended Solids (TSS), which reached 14.467 g L^−1^ compared with 96 g L^−1^ in our study (6.6‐fold higher). Although we evaluated the ability of the bacterial isolates to coexist in a consortium (data not shown), most strains were unable to grow together, with the exception of OWB‐42 and OWB‐43. However, considering that OWB‐42 was not able to significantly reduce the COD parameter when evaluated individually, the bioremediation potential of these isolates as a consortium was not assessed.

Electrical conductivity in the optimised mix was 13.50 dS m^−1^, considerably higher compared to that of dairy industry wastewater (0.35 dS m^−1^) (Tikariha and Sahu 2014), but similar to olive mill wastewater stored in open‐air ponds (Martínez‐Gallardo et al. 2021; Haouache and Bouchaleta 2016). EC is a critical parameter for soil disposal, due to its influence on nutrient availability in the root zone and osmotic pressure (Ding et al. 2018). These authors reported that the optimal range for maximum yield was between 2.0 and 2.5 dS m^−1^. While some bacterial strains, such as *B. subtilis* OWB‐10, *B. velezensis* OWB‐30, and *P. aeruginosa* OWB‐41, reduced EC, *Cytobacillus* sp. OWB‐43 did not. Considering the dilution factor applied to the sample, the results suggest that, in addition to EC, another factor negatively affecting lettuce development was reduced after treatment with OWB‐43.

A marked reduction in residue phytotoxicity was observed following bacterial treatment, with significant improvements in growth parameters in both lettuce seedlings and onion bulbs used as biosensors, under both agitated and static growth conditions. Soil irrigated with *Cytobacillus* sp. OWB‐43‐treated residue supported lettuce growth matching values of fresh and dry weight seen with water irrigated soil controls. Considering both residue treatment conditions—bacterial growth under agitation or static culture—similar results were obtained in lettuce hypocotyl elongation, while for root elongation greater growth was observed when the residue was treated under agitation. Nevertheless, comparing onion bulbs root elongation growing in soil irrigated with the treated residue in static conditions, 68% of the length of controls grown in water was observed, whereas treatment under agitation showed values of 51%, putting forward that static treatment was more effective in reducing phytotoxicity, besides its advantage in less energy use.

NMR analysis is a powerful analytical technique widely used for both the quantification and identification of chemical compounds (Masotti et al. 2021), although limited by signal overlap. Our NMR analysis supported the occurrence of chemical alterations in several compounds present in treated OWM, some of which were likely responsible for phytotoxicity. Notably, acetate and glycerol were depleted during treatment, indicating their use as carbon sources. Acetate, a simple organic acid, serves as a readily available carbon source for many bacteria due to their ability to metabolise it through various biochemical pathways (Schilling et al. 2007; Pinhal et al. 2019). Glycerol is imported into the cell through specific transport systems, which allows bacteria to utilise it efficiently when it is available in the environment (Vikromvarasiri et al. 2023; Kalantari et al. 2017). The metabolic ability to adapt to different environmental conditions makes the family Bacillaceae significant in both ecological contexts and biotechnological applications (Harirchi et al. 2022). Bioautography assays suggested the presence of several phytotoxic compounds and phytotoxicity was consistently lower in samples treated with *Cytobacillus* sp. OWB‐43. Overall, these results support the feasibility of bioguided isolation of phytotoxic compounds in future studies.

Bioremediation processes under static conditions had previously proven effective in treating dye manufacturing wastewater (Patel et al. 2015; Haque et al. 2020) and mineral transformed oils contaminated with polychlorinated biphenyls (Sadañoski et al. 2020). Furthermore, under static conditions some bacteria were capable of forming biofilms at liquid‐air interfaces without the need for an additional support or filter on which to form the structures (O'Toole et al. 2000). Such was the case of *B. cereus*, *B. subtilis*, *P. fluorescens* and *Acinetobacter* species, among other previously studied bacteria (Constantin 2009; Martí et al. 2011). In bioremediation, biofilms presented a more efficient and safer alternative than bioremediation with planktonic microorganisms, since cells in the biofilm had better adaptation and survival (Constantin 2009; Piazza et al. 2019). In addition, as a result of the physical and physiological interactions among the organisms within the biofilms, the use of xenobiotics was enhanced, showing an advantage when used in industrial plants for immobilisation and degradation of contaminants (Singh et al. 2006; Mishra et al. 2022). To evaluate the potential scalability and industrial applicability of the process, experiments were conducted under static conditions. *Cytobacillus* sp. OWB‐43 significantly reduced its phytotoxicity, enabling safe soil disposal. The lower energy requirements associated with bacterial growth in static systems suggest that this strategy may be more feasible for large‐scale applications. However, further studies are required to more thoroughly evaluate the scalability of the process. It is likely that an aeration system will be necessary in the treatment tanks to ensure adequate oxygen supply and maintain optimal microbial activity. Therefore, the long‐term accumulation of untreated olein waste in open‐air pools could potentially be overcome by this biotechnological approach, which may also serve as a foundation for future studies focused on olein waste treatment. The wastewater used in this study is classified as non‐hazardous industrial waste. However, even after bacterial treatment with strain OWB‐43, the COD levels remained too high for discharge into sewage systems or natural waterways. Nevertheless, the treated wastewater could be safely disposed of on land. This work highlights the potential of bacteria isolated from industrial waste for developing sustainable bioremediation strategies. Further large‐scale research is needed to assess its practical applicability.

## 5 Conclusions

The isolated strain *Cytobacillus* sp. OWB‐43, obtained from olein industrial waste, demonstrated the ability to grow in optimised olein waste under both agitation and static conditions. Its application significantly reduced the phytotoxicity of the waste, enabling its safe disposal on soil. Our approach not only highlights the potential of targeted microbial treatment for effective bioremediation of industrial residues but also contributes to the development of sustainable strategies for waste management.

## Author Contributions

**Julián Escalante:** conceptualization, investigation, writing – review and editing. **Nicolás Lencina:** conceptualization, investigation. **Ainelén Piazza:** conceptualization, investigation, writing – review and editing. **Paula Burdisso:** validation, writing – review and editing. **Mario O. Salazar:** writing – review and editing, investigation. **Ricardo L. E. Furlan:** writing – review and editing, conceptualization. **Natalia Gottig:** conceptualization, writing – review and editing. **Jorgelina Ottado:** writing – review and editing, conceptualization. **Betiana Garavaglia:** writing – original draft, conceptualization, supervision, writing – review and editing, funding acquisition.

## Funding

This work was supported by Grants from Agencia Nacional de Promoción Científica y Tecnológica (PICT2020‐0412 to BSG) and Agencia Santafesina de Ciencia, Tecnología e Innovación (IO2017‐00011 to BSG). JE and NL are fellow and PB, MS, RF, JO, NG and BSG are staff members of Consejo Nacional de Investigaciones Científicas y Técnicas (CONICET, Argentina).

## Conflicts of Interest

The authors declare no conflicts of interest.

## Supporting information


**Figure S1:** Schematic representation of steps required for the edible oil refinement and subsequent generation of the waste used in this work, acid water (AW) and oily interface (OI). Adapted figure (Rajkumar et al. 2010).

**Figure S2:** Schematic representation of the isolation protocol used.

**Figure S3:** Bacterial growth. Growth of *B. subtilis* OWB‐10 (OWB‐10), *B. velezensis* OWB‐30 (OWB‐30), *P. aeruginosa* OWB‐41 (OWB‐41), *Lysinibacillus* sp. OWB‐42 (OWB‐42), *Cytobacillus* sp. OWB‐43 (OWB‐43), *Enterobacter* sp. OWB‐48 (OWB‐48) and *Niallia* sp. OWB‐49 (OWB‐49) was evaluated in LB media on 96‐wells microplate at 30°C. Bacterial growth rates were calculated and shown in the table.

**Figure S4:** Taxonomic classification made by MiGA webserver.

**Figure S5:** Quantification of electrical conductivity (EC) (dS m^−1^) tested for *B. subtilis* OWB‐10 (OWB‐10), *B. velezensis* OWB‐30 (OWB‐30), *P. aeruginosa* OWB‐41 (OWB‐41), *Lysinibacillus* sp. OWB‐42 (OWB‐42), *Cytobacillus* sp. OWB‐43 (OWB‐43), *Enterobacter* sp. OWB‐48 (OWB‐48) and *Niallia* sp. OWB‐49 (OWB‐49). Values represent the means of three samples and are representative of three independent experiments. Error bars are standard deviations.

**Figure S6:** Evaluation of mobile phases polarities for separation of aqueous fraction obtained from untreated OWM (C) or OWM treated with *Cytobacillus* sp. OWB‐43 (T). Representative images of plates developed at different wavelengths (254, 302, and 365 nm) are shown for the different mobile phases. (A) chloroform: ethyl acetate: acetic acid (10:10:1); (B) methanol: acetic acid (19:1); (C) methanol: water: acetic acid (10:10:1); (D) absolute ethanol and (E) ethanol: water (85:15).

**Figure S7:** Thin layer chromatography (TLC) separation and bioautographic phytogrowth‐inhibitory bioassay (BPIB). TLC plate diagram after separation, numbers indicate the regions into which it was divided for later evaluation (A). Box plots corresponding to the hypocotyl and root length of the seedlings obtained onto TLC plate after incubation for each treatment and region. BPIB using absolute ethanol as mobile phase for chromatographic separation was performed for the untreated sample and OWM inoculated with *Cytobacillus* sp. OWB‐43. As experimental control, TLC maintained under same conditions without sample seeding was evaluated (B). Conditions that presented statistically significant differences are indicated with asterisks: *p < 0.05, **p < 0.01, ***p < 0.001.

**Figure S8:** Bacterial growth on agar plate. Representative images of isolated strains plated onto (A) 2% (w/v) agar plate prepared with olein waste diluted by half in tap water (OWM:H_2_O) with pH regulated with building lime and (B) without pH regulation. Incubation time: 7 days at 30ºC.

**Table S1:** strains genome assembly data.
**Table S2:** Physicochemical parameters of conditioned OWM:H_2_O.
**Table S3:** average values of hypocotyl and root length from TLC regions evaluated by the bioautographic phytogrowth‐inhibitory bioassay (BPIB), for the untreated sample (control) or OWM:H_2_O treated with *Cytobacillus* sp. OWB‐43 (OWB‐43). Statistical significance of the differences between samples after performing the corresponding analyses. Conditions that showed statistically significant differences are indicated with asterisks: *p < 0.05, **p < 0.01, ***p < 0.001. Mobile phase: absolute ethanol.

## Data Availability

Information about *B. subtilis* OWB‐10, *B. velezensis* OWB‐30, *P. aeruginosa* OWB‐41, and *Cytobacillus* sp. OWB‐43 sequencing is provided in https://www.ncbi.nlm.nih.gov/bioproject/1053253.
