# Supplementary material for: Bioremediation of Olein Industry Waste by Cytobacillus sp. OWB‐43 Strain
Source: Environ Microbiol Rep. 2026 Oct 8;18(5):e70424. doi: 10.1111/1758-2229.70424 (PMC13649369; doi:10.1111/1758-2229.70424)
Supplement: Supplementary file 1 [file EMI4-18-e70424-s008.docx]

**Supplementary Table 1**: strains genome assembly data

| ***Isolated Strain*** | ***Contigs*** | ***N50 value (bp)*** | ***Total length (bp)*** | ***Longest sequence (bp)*** | ***G+C content (%)*** |
| --- | --- | --- | --- | --- | --- |
| **OWB-10** | 24 | 454,725 | 4,066,369 | 717,682 | 43.6434 |
| **OWB-30** | 21 | 357,763 | 4,178,377 | 1,089,686 | 45.8884 |
| **OWB-41** | 65 | 428,511 | 6,473,911 | 1,022,891 | 66.3418 |
| **OWB-43** | 24 | 578,758 | 4,471,664 | 2,002,860 | 36.7698 |

**Supplementary Table 2**: Physicochemical parameters of conditioned OWM:H_2_O

| **Parameter** | **Optimized olein waste mix (OWM) diluted in tap water** |
| --- | --- |
| **pH** | 7.0 ± 0.2 |
| **Total solids (g L^-1^)** | 96 ± 2 |
| **COD (mg L^-1^)** | 160,200 ± 5,500 |
| **SSE (g L^-1^)** | 29.0 ± 1.7 |
| **EC (dS m^-1^)** | 13.5 ± 0.4 |
| **Sulfate (g L^-1^)** | 9.2 ± 0.8 |
| **Total Nitrate (mg L^-1^)** | 150 ± 13 |
| **Total Nitrite (mg L^-1^)** | undetectable |
| **Total Chloride (g L^-1^)** | 2.4 ± 0.2 |
| **Fe 57 (mg L^-1^)** | 289 ± 8 |
| **Zn 66 (mg L^-1^)** | 3.2 ± 0.1 |
| **P 31 (g L^-1^)** | 2.40 ± 0.03 |
| **Na 23 (g L^-1^)** | 2.02 ± 0.05 |
| **K 39 (mg L^-1^)** | 976 ± 30 |
| **Mg 24 (mg L^-1^)** | 629 ± 18 |
| **Ca 43 (g L^-1^)** | 7.59 ± 0.22 |
| **Al 27 (mg L^-1^)** | 157 ± 5 |
| **Heavy metals** | undetectable |

**Supplementary Table 3**: average values of hypocotyl and root length from TLC regions evaluated by the bioautographic phytogrowth-inhibitory bioassay (BPIB), for the untreated sample (control) or OWM:H_2_O treated with *Cytobacillus* sp. OWB-43 (OWB-43). Statistical significance of the differences between samples after performing the corresponding analyses. Conditions that showed statistically significant differences are indicated with asterisks: * *p < 0.05*, ** *p < 0.01*, *** *p < 0.001*. Mobile phase: absolute ethanol.

| **Region** | **Hypocotyl length (mm)** | | ***p-value*** | **Root length (mm)** | | ***p-value*** |
| --- | --- | --- | --- | --- | --- | --- |
|  | **Control** | **OWB-43** |  | **Control** | **OWB-43** |  |
| **1** | 6.0 | 8.4 | **0.006 | 5.8 | 10.3 | **0.004 |
| **2** | 8.6 | 8.9 | 1.00 | 8.9 | 12.0 | 0.87 |
| **3** | 8.5 | 8.0 | 1.00 | 9.0 | 13.0 | 0.91 |
| **4** | 7.9 | 9.1 | 1.00 | 8.1 | 12.1 | 0.17 |
| **5** | 7.3 | 8.7 | 1.00 | 7.8 | 9.7 | 1.00 |
